# In-depth investigation into the binding capabilities of palladium-based N-heterocyclic carbenes towards five heavy metals: Hg, Cd, Pb, Cr, and As

**DOI:** 10.1371/journal.pone.0330984

**Published:** 2025-09-26

**Authors:** Qiao Liu, Xianghe Kong, Qian Ru Li, Wenbo Lan

**Affiliations:** 1 School of Chemical Biology and Environmental Engineering, Xiangnan University, Chenzhou, Hunan, China; 2 School of Chemistry and Chemical Engineering, University of South China, Hengyang, Hunan, China; 3 School of Public Health, Xiangnan University, Chenzhou, Hunan, China; Kwara State University, NIGERIA

## Abstract

Given the severe environmental and health risks posed by heavy metal pollutants such as mercury (Hg), cadmium (Cd), lead (Pb), chromium (Cr), and arsenic (As), the development of highly efficient and selective capture materials is critically important.This study explores the molecular structural characteristics of complexes formed by substituting the central palladium atom in nitrogen-heterocyclic carbene complexes with mercury (Hg), cadmium (Cd), lead (Pb), chromium (Cr), and arsenic (As). For all the complexes, the simulated infrared and ultraviolet-visible spectra, Wiberg bond indices, as well as the binding ability of nitrogen-heterocyclic carbene ligands to the central atom were assessed. The results show that the complexes obtained when the central atom is replaced by Hg, Cd, Pb, Cr, or As are stable and display unique spectral features. Significantly, nitrogen-heterocyclic carbene has the strongest binding affinity for Pd. The complexes of chromium substitutes possess relatively high chemical stability. These findings will offer important references for future pollution control and support the specific capture of the five studied heavy metals, and enrichment of Cr using nitrogen-heterocyclic carbene ligands.

## 1. Introduction

Heavy metals and their compounds exhibit stable chemical properties, making them difficult to decompose in the environment. As a result, they can accumulate over extended periods, posing significant risks to human health upon exposure [1]. Once introduced into the human body, these metals often accumulate in tissues to varying degrees, leading to toxic effects. Mercury (Hg) accumulation primarily inflicts harm on the nervous system and damages brain function [2–4], resulting in mercury poisoning encephalopathy characterized by symptoms such as limb numbness and diminished visual and tactile sensations [5]. In severe cases, this condition may lead to death due to heart failure.The mercury cycle serves as a quintessential example of a heavy metal ecosystem cycle. Mercury undergoes movement and transformation within water, soil, atmosphere, and food chains in its elemental state; its compounds and vapor are predominantly highly toxic [6]. Cadmium (Cd), a non-essential metal element in the human body, is capable of causing both acute and chronic toxicity [7,8]. Acute cadmium poisoning is marked by acute gastrointestinal irritation, whereas chronic cadmium poisoning presents as an inhibition of macrophage phagocytosis [9]. The accumulation of cadmium within the body can lead to the formation of urinary calculi and renal impairment [10,11]. Furthermore, cadmium poisoning can result in cerebral inflammation, metabolic disturbances in the brain, hypertension, and histopathological alterations in the testes and ovaries [12]. Lead (Pb) is the non-radioactive element with the largest atomic weight, and its accumulation in the human body usually leads to acute and chronic poisoning. Acute poisoning commonly presents as gastrointestinal irritation symptoms, accompanied by a metallic oral taste, abdominal pain, headache and other phenomena [13]. Chronic lead poisoning may impair renal tubular reabsorption function, cause micro-artery spasms and thrombosis, cause reproductive dysfunction, cause liver enlargement, jaundice, cirrhosis, and even liver necrosis. Cadmium (Cd), which is a non-essential metal element in the human body, can cause both acute and chronic toxicity [14,15]. Chromium (Cr) accumulates mainly in the liver, kidneys and lungs, and may lead to liver tissue degeneration, central vascular necrosis, renal tubular necrosis and pneumonia [16,17]. Hexavalent chromium (Cr (VI)) compounds are able to cause mutations in the DNA structure and have carcinogenic and genetic effects. The genetic effects can influence the intellectual development of infants [18]. In soil, the accumulation of Cr (VI) causes Cr ions to bind to proteins in plant cells, thus hindering crop growth and leading to a loss of cell activity [19,20]. Arsenic(As) belongs to metalloids and is a Class I carcinogen. It has multiple valence states in nature and is found in two forms: organic arsenic and inorganic arsenic. Its toxicity is closely related to the valence state and the form of existence. Normally, As3 + has the strongest toxicity [21]. The toxicity of inorganic arsenic is higher than that of organic arsenic [22]. Arsenic is associated with the occurrence and development of various diseases involving multiple organs and systems throughout the body. Arsenic exposure is closely related to diseases such as atherosclerosis, cardiovascular diseases, and cancers. Clinically, acute arsenic (As) poisoning commonly presents with acute gastrointestinal symptoms [23]. Arsenic can damage gastrointestinal mucosal cells through related oxidative stress and inflammatory responses, disrupt the intestinal barrier, and promote the occurrence and development of gastric cancer [24]. It can also directly trigger inflammatory responses and damage the gastrointestinal epithelium. Arsenic poisoning can cause liver diseases and present a spectrum of characteristics, including simple liver injury, fatty liver, fibrosis, and even liver cancer [25]. Arsenic can also indirectly affect the content of trace elements and amino acids in the human body by affecting the nutrient elements of rice [26].

There are two commonly used methods for detecting lead. One is atomic absorption spectroscopy (AAS), which is based on the absorption characteristics of lead atoms for specific wavelengths of light [27]. It quantifies the lead by digesting the sample and measuring the absorption. The alternative method is inductively coupled plasma mass spectrometry (ICP-MS) [28,29]. In this approach, the sample is digested, ionized by an inductively coupled plasma, and then the lead ions are detected by a mass spectrometer based on the mass-to-charge ratio. Mercury has a low boiling point, and its determination mainly employs cold atomic absorption spectroscopy and atomic fluorescence spectroscopy (AFS) [30,31]. Cold atom absorption spectroscopy exploits the strong absorption of light by mercury atoms at specific wavelengths. It measures the absorbance by reducing mercury ions to mercury atoms with a reducing agent. AFS is commonly used to reduce mercury ions to mercury atoms with potassium borohydride. The mercury atoms are excited to produce fluorescence and the mercury content is quantified by detecting the fluorescence intensity. There are two commonly-used atomic absorption methods for cadmium measurement, namely the flame atomic absorption spectroscopy method and the graphite furnace atomic absorption spectroscopy (GFAAS) method [32–34]. The flame atom absorption method uses a reducing gas to reduce and atomize a sample at extreme temperatures and then measures the absorbance at the characteristic wavelength of cadmium to determine its content. GFAAS atomizes the sample using the elevated temperature in a graphite furnace, which can effectively increase the atomization efficiency and the detection limit. Arsenic is a metalloid and is one of the so-called “five toxic heavy metals”. There are two main approaches to its detection. One is the silver salt method [35]. In this method, the arsenide is first reduced to arsine gas, which reacts with silver nitrate to form a yellow precipitate. Then, it is quantified by the colorimetric method [36]. Another method is atomic fluorescence spectroscopy. In this method, arsenic ions are first reduced to arsenic gas using potassium borohydride, and then they are excited to produce fluorescence. The arsenic content was quantified by detecting the fluorescence intensity.

N-Heterocyclic carbene (NHC) is a neutral two-electron donor capable of bonding with various metals. Its properties are similar to those of organophosphine ligands, and it is more easily synthesized and functionalized than organophosphine ligands. When NHC bonds with metals, the resulting C-M bond is stronger than the P-M bond [37]. Metal NHC complexes are primarily used as catalysts in chemical reactions [38,39]. In recent years, transition metal (silver, gold, rhodium, and platinum, etc.) NHC complexes have demonstrated promising anti-tumor activity by inhibiting tumor cell proliferation, and different metal centers often exhibit different mechanisms of action. N-Heterocyclic carbenes (NHCs) are compounds composed of nitrogen atoms and heterocyclic structures. Nitrogen atoms typically carry one lone pair of electrons, making NHCs strong nucleophiles and electron donors. The divalent carbon atoms in NHCs are located on top of these ring structures and are connected by covalent bonds to nitrogen atoms and two different groups, forming a deeply conjugated system [40]. When acting as a ligand, NHCs form conjugated systems with rigid planar structures, and NHC metal complexes can absorb UV light and emit significant fluorescence. Consequently, the characteristics of NHC metal complexes can be gauged through ultraviolet-visible (UV-Vis) absorption and fluorescence.As a transition metal, palladium (Pd) has been deeply studied and fully characterized in the field of catalysis due to the excellent stability and well-defined planar geometry of its NHC complexes (Pd-NHC) [41]. This structural feature makes Pd-NHC an ideal benchmark model for investigating the interaction between NHC ligands and metals. NHC ligands have coordination cavities where divalent carbon and nitrogen atoms at the coordination center can bind to heavy metal ions. When the palladium atom at the NHC center is substituted by Hg, Cd, Pb, Cr, or As, the UV-Vis (ultraviolet-visible) absorption spectra of the newly formed complexes are prone to variation. Considering the substantial potential of copper, nickel, and cobalt compounds in catalytic applications, particularly with respect to sustainability; the regulation and utilization of photophysical properties of cost-effective complexes; and the significant value of these metal complexes in the fields of biology and medicine, scholars have conducted extensive research on N-heterocyclic carbene (NHC) complexes of copper, nickel, and cobalt, achieving important advancements [42], However, research on N-heterocyclic carbene complexes associated with heavy metal ions such as Hg, Cd, Pb, Cr, and As remains relatively limited.Density functional theory (DFT) is commonly employed for the simulation and prediction of metal complexes [43], and research has indicated that its computational outcomes are extremely in line with experimental results. Occasionally, DFT calculations can replace traditional experiments [44]. While DFT calculations have been used to analyze N-heterocyclic carbenes and their complexes, the use of DFT in studies involving spectroscopic analysis of NHC ligands bound to heavy metals is less common, providing a reference for heavy metal detection.

## 2. Research methods

In this article, all DFT (density functional theory) calculations were carried out with the Gaussian 16 package by means of the DFT/B3LYP [45] hybrid functional. For Pb (lead), Hg (mercury), Cd (cadmium), Cr (chromium), or As (arsenic), the small-core relativistic pseudopotential ECP28MDF was utilized, and for C (carbon), O (oxygen), N (nitrogen), and H (hydrogen), the 6-311G basis set was applied [46,47]. In the gas-phase symmetric case, the optimized complex molecular structure is calculated at the LanL2MB/6-311G theoretical level without symmetry constraints. After all molecules were structurally optimized, the vibrational frequencies were calculated for validation. Provided that no imaginary frequencies were detected in the vibrational frequencies, and under the same groups and conditions, the Mulliken charges and Wiberg bond indices (WIBs) of the optimized structures were calculated. The time-dependent density functional theory (TD-DFT) method [48] was utilized to calculate the gas-phase ultraviolet-visible spectra. Moreover, the same TD- DFT method was used to determine electronic properties, such as the energies of the highest occupied molecular orbital (HOMO) and the lowest unoccupied molecular orbital (LUMO). The results were carefully analyzed to elucidate the electronic transitions responsible for the observed spectral features.

## 3. Results and analysis

### 3.1 Molecular structure

Coordination bond is a type of covalent bond formed between an organic ligand and metal atoms or ions. Its bond energy lies between that of a hydrogen bond and a covalent bond [49]. The length of a coordination bond can directly reflect the strength of the chemical bond formed between two atoms [50]. However, for certain rigid ligand molecules, because of differences in the radius of the central atom and the characteristics of the formed complexes, the bond length alone might not fully reflect the coordination ability. Thus, by combining bond angles with bond lengths, a comprehensive understanding of the coordination ability of rigid ligand molecules with the central atom can be obtained. Specifically, through analyzing the bond angles of coordination bonds, we can evaluate the strength of the interatomic interactions between the rigid ligand molecule and the central atom more accurately, thereby making a more precise judgment of the coordination ability. This comprehensive analysis method takes into account not only the effect of bond length but also incorporates the important parameter of bond angle, providing us with a deeper understanding of chemical bonding.

**Fig 1:**
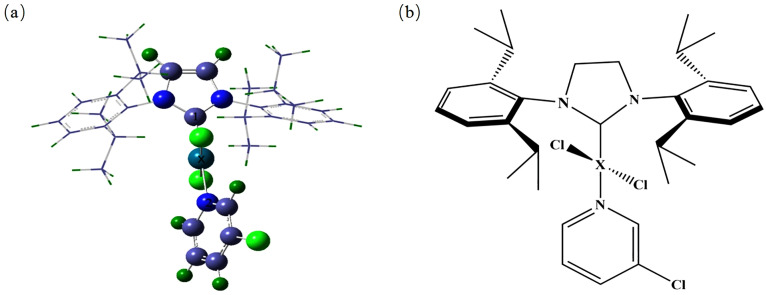
The optimized N-heterocyclic carbenes.

As illustrated in Fig 1, complexes formed between N-heterocyclic carbenes and a variety of heavy metal elements display analogous structural features. Fig 1(a) presents the optimized N-Heterocyclic carbenes, Fig 1(b) shows a schematic representation in the ChemDraw style of a typical NHC complex.In this structure, the central atom “X” represents the Pd atom in the N-heterocyclic carbene molecule. In the palladium complexes of heavy metals with N-heterocyclic carbenes, the Pd atom can be replaced by Pb, Hg, Cr, Cd, or As to form Pb, Hg, Cr, Cd, and As complexes, respectively. Certain specific atoms in these complexes play critical roles, which is why they have been numbered in Fig 1. After detailed optimization of these complex structures, we obtained the main structural parameters, including bond lengths and bond angles, with specific data presented in Table 1. These structural parameters provide key information for a deeper understanding of the properties and behaviors of these complexes.

**Table 1:** The important bond lengths (nm), bond angles (°)of the optimized complexes compared with the N-heterocyclic carbenes.

Complex	Complex-Pd	Complex-Hg	Complex-Cd	Complex-Pb	Complex-Cr	Complex-As
C1-X	2.03026	2.337	2.29687	2.39738	2.03498	2.17079
N2-X	2.12648	2.44605	2.33503	2.43471	1.9817	2.5357
C1-X-N2	179.84691	124.18318	121.88168	101.82753	115.87168	155.78479

In N-heterocyclic carbene-heavy metal complexes, the bond lengths between the central atom and the ligand atoms C1 and N2, when the central atom is Pb, Hg, Cd, or As, are all longer than the C-Pd bond length. Specifically, when the central atom is replaced by Cr, the coordination bonds in the cavity structure elongate. The coordination bonds in other N-heterocyclic carbene heavy metal complexes are also longer than those in N-heterocyclic carbene palladium, primarily due to the central atom deviating from the position of Pd. That is to say, the atomic radii of arsenic (As), cadmium (Cd), mercury (Hg) and lead (Pb) are larger than that of palladium (Pd). When these atoms replace the central Pd atom, the central atom will deviate from the plane where the four coordinating atoms are located. Consequently, the bond lengths containing nitrogen (N) atoms in the coordination bonds formed by As, Cd, Hg, and Pb are longer than those of N-Pd. These observations suggest that the atomic radius of the central atom has a significant influence on the length of the coordination bonds. An increase in the atomic radius results in the elongation of the coordination bonds, thereby affecting the overall structure and properties of the complex. This finding is of great significance for understanding the chemical behavior of heavy-metal complexes and designing new catalysts.

### 3.2 WIBs

In this article, the calculation of Wibs is based on the results of Natural Bond Orbital (NBO) analysis [51], as shown in Table 2, where X corresponds to Pd, Pb, Hg, Cr, Cd, or As atoms. In our study of N-heterocyclic carbene palladium, we discovered that the Wiberg Index value of the C1-Pd bond is 0.4789, whereas that of the N2-Pd bond is 0.3135. For C bonds formed by other heavy metal central atoms, the Wiberg Index values vary from 0.2805 to 0.8497, and for N bonds, the values range from 0.3135 to 0.6274. An important finding is that the Wiberg Index values of the N-Cr bond and the C-Cr bond reach the highest values of 0.6274 and 0.8497,respectively, which indicates that the bonding strength between Cr atoms and other atoms is particularly remarkable in these systems [52].

**Table 2:** Wiberg bond indices of the heavy metal complexes.

Complex	Complex-Pd	Complex-Hg	Complex-Cd	Complex-Pb	Complex-Cr	Complex-As
C1-X	0.4789	0.6673	0.661	0.7086	0.8497	0.2805
N2-X	0.3135	0.3826	0.401	0.4697	0.6274	0.0359

This data analysis is based on the measurement of bond length between two atoms in all complexes. Due to the rigid structure of N-heterocyclic carbene, significant differences are presented in the length of coordination bonds. When the central atom Pd is replaced by heavy metal toxic atoms, a noticeable increase in bond values can be observed. This increase may be attributed to the enhanced electron-sharing ability between the coordinated nitrogen and carbon atoms and the central atom in the newly formed complexes. Meanwhile, this enhancement leads to a decrease in the positive charge of the central atom and the negative charge of the coordinated nitrogen atom.

The improvement of electron-sharing ability can be ascribed to the high electron density and strong electronegativity of heavy metal atoms. These properties allow the electron clouds to overlap more effectively with the coordinated nitrogen and carbon atoms, thereby strengthening the chemical bonds. In addition, the introduction of heavy-metal atoms may alter the electron distribution within the coordination environment, thus optimizing the electronic structure of the coordination bonds. This adjustment results in a reduction in the positive charge of the central atom and the negative charge of the coordinated nitrogen atom, ultimately achieving a re-balanced charge distribution.

### 3.3 Infrared spectrogram

**Fig 2:**
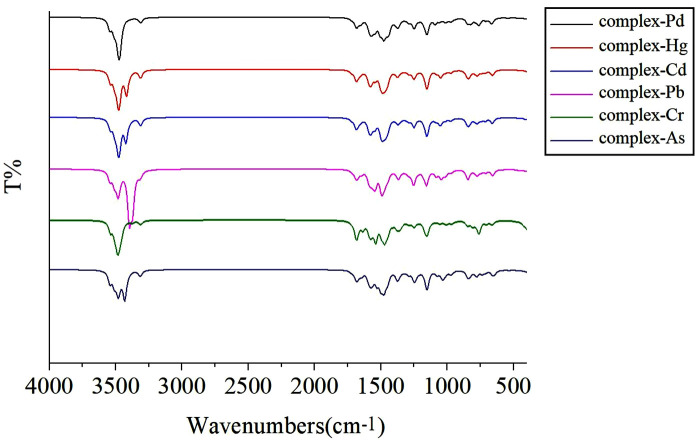
Simulated infrared spectra of the heavy-metal complexes.

In this study, we carried out a comprehensive simulation analysis of the infrared spectrum (IR) and the vibrational spectrum of the optimized complex by using consistent computational strategies and computer clusters. Specifically, the infrared spectrum data of the complex are presented in Fig 2. The analysis results show that no imaginary frequencies were detected within the vibration frequency range of all the examined complexes, a finding that strongly confirms that the structure of the studied complexes has reached the optimal state of stability. Further vibration analysis clearly indicates that when the central atom of the N-heterocyclic carbenes is substituted by heavy metal atoms such as Pd, Pb, Hg, Cr, Cd, or As, significant changes occur in the infrared characteristic peaks of the coordination bond. The differential changes in these infrared absorption peaks will provide crucial reference information for the qualitative analysis of complexes formed by N-heterocyclic carbenes with different heavy metals in future experiments. For convenience of reference, Table 3 lists in detail the characteristics of the important vibrational absorption peaks shown in Fig 2.

**Table 3:** The representative IR data (cm^-1^) for the complexes.

Complex	νC-H	νC = N	Vibration absorption peak of the central pyrazole skeleton
Complex-Pd	3545、3511、3487	1542、1506、1483	1434
Complex-Hg	3540、3507、3485	1542、1502、1488	1374.5
Complex-Cd	3540、3507、3485	1501、1487	1370
Complex-Pb	3543、3510、3483	1542、1498、1490	1366
Complex-Cr	3539、3505、3487	1636、1538、1494	1379
Complex-As	3543、3511、3433	1530、1501	1357

After various atomic substitutions for the central atom, the positive charge of the central metal atom decreases. In contrast to palladium (Pd), the introduction of heavier central atoms such as mercury (Hg), cadmium (Cd), and lead (Pb) results in a blue shift in the C = N bond stretching frequency, ranging from 4 to 41 cm^-1^. Conversely, when chromium (Cr) is positioned as the central atom, a red shift ranging from 11 to 94 cm-1 is detected in the C = N bond. It is noteworthy that, When Pb acts as the central element in the formation of complexes, its vibration peak is conspicuously enhanced, which is potentially attributed to the stretching vibration of functional groups.

When cadmium, lead or arsenic replaces Pd to occupy the central position in the complex, an obvious blue-shift phenomenon will occur in the C-H stretching vibration absorption peak value. Compared with palladium (Pd), the vibration absorption peaks related to these substitute central atoms decrease, with the decrease ranging from 3 to 46 cm^-1.^ More importantly, replacing the central atom in N-heterocyclic carbene with various heavy metal atoms will not only affect the electronic properties but also cause structural changes in the resulting complexes. This structural change is a crucial factor that must be considered when the overall stability and reactivity of the complexes are taken into account.

### 3.4 Charge distribution

The natural electrostatic charge distributions between the ligand atoms and the central metal in N-heterocyclic carbene Pd and other complexes formed by Pd substitution are shown in Table 4.

**Table 4:** The charge distributions of the heavy-metal complexes.

Complex	C	N	X
Complex-Pd	0.25082	−0.11068	0.04821
Complex-Hg	0.28451	−0.13842	0.28451
Complex-Cd	0.37578	−0.15554	0.37578
Complex-Pb	0.5282	−0.17000	0.5282
Complex-Cr	0.07593	−0.15193	0.07593
Complex-As	0.27397	−0.12665	0.27397

It can be seen from the electrostatic charge distribution values in Table 4 that, in N-heterocyclic carbenes, unlike palladium, the substitution of the central atom with elements such as Hg, Cd, Pb, Cr, or As increases the absolute value of the negative charge on the coordinating nitrogen atoms. This change in charge distribution is significant, indicating that after the central atom is substituted for palladium, the electron density provided by the coordinating atoms to the central metal atom is reduced.

This result also indicates the crucial role of the central atom in influencing the structure and electronic properties of the coordination bonds. When other metal ions replace palladium, it directly affects the electron-sharing ability between the coordinating atoms and the central metal atom, thereby enhancing the overall electron density distribution within the complex. This phenomenon can greatly enhance the understanding of the stability of the complex.

The conclusions reached in this analysis are in agreement with the results acquired via the WIBs method, further confirming the reliability of the charge distribution analysis. This consistency in methodology not only verifies the findings but also underlines the importance of carefully choosing central metal atoms in N-heterocyclic carbene complexes so as to optimize their electronic and structural characteristics.

### 3.5 Ultraviolet-visible absorption spectroscopy

**Fig 3:**
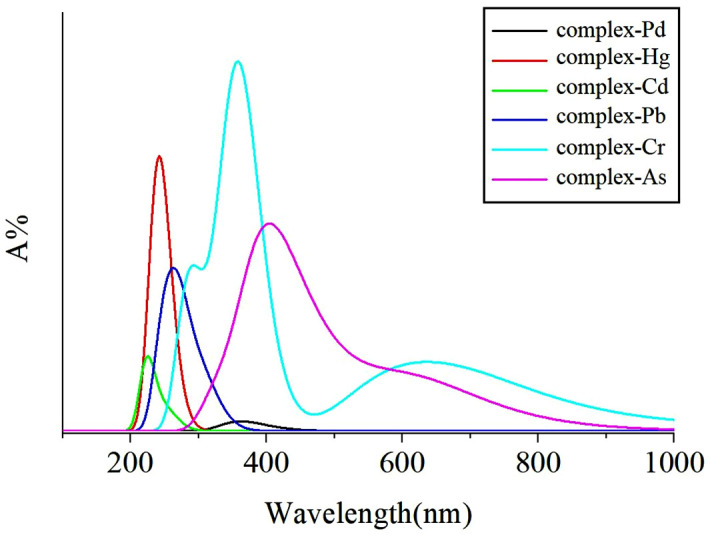
Simulated ultraviolet visible absorption spectra for the heavy-metal complexes.

In a vacuum environment, a detailed study was conducted on the ultraviolet-visible absorption spectra of N-heterocyclic carbene palladium and its related heavy metal complexes, the results of which are shown in Fig 3. The study covered N-heterocyclic carbene palladium, mercury complexes, cadmium complexes, lead complexes, chromium complexes, and arsenic complexes. The ultraviolet-visible absorption peaks of these complexes were located at 362.8 nm, 240.4 nm, 224.2 nm, 263.8 nm, 357.4 nm, and 404.2 nm, respectively.

The research results indicate that when the palladium atom is replaced by mercury, cadmium, or lead atoms, the ultraviolet-visible absorption peaks of the formed complexes exhibit a blue shift. This phenomenon may be attributed to the larger positive charge of the mercury, cadmium, and lead atomic nuclei, which produces a stronger electron withdrawal effect, leading to a blue shift in the ultraviolet absorption spectrum. In contrast, when the central atom is arsenic, the ultraviolet-visible absorption peaks of the complexes undergo a red shift, and these absorption peaks are all within the visible spectrum range.

Further analysis shows that the relative absorbance of N-heterocyclic carbene palladium is significantly lower than that of other heavy metal complexes formed with N-heterocyclic carbene. Notably, although the chromium complex experienced only a slight red shift (5.4 nm), its ultraviolet absorption range was broader than that of N-heterocyclic carbene palladium. Therefore, through ultraviolet-visible absorption spectra, it is possible to definitely distinguish the N-heterocyclic carbene palladium from chromium complexes formed in the experiment. Interestingly, the complexes formed by other metal ions exhibit almost no absorption peaks within the range of 600–800 nm. However, As and Cr possess distinct and relatively broad absorption peaks. This could be attributed to the significantly smaller atomic radii of As and Cr compared to the other three metal ions. During the formation of complexes, the delocalization of the electron cloud is relatively stronger, enhancing the stability of the complex molecules (which is in accordance with the binding energy data presented in Table 5), and possibly enabling the complexes to display absorption peaks of a certain intensity in the visible light region. This finding is of significant importance for understanding the spectral characteristics of different heavy metal complexes and their applications in materials science and chemistry.

**Table 5:** Binding energies formed between various heavy metal ions and N-heterocyclic carbene ligands (kJ/mol).

Complex	Complex-Pd	Complex-Hg	Complex-Cd	Complex-Pb	Complex-Cr	Complex-As
Binding energy	−561.88	−321.85	−352.41	−373.05	−480.61	−409.61

### 3.6 Binding energy

The molecular binding energy serves as a critical parameter for evaluating the affinity of ligands toward the central atom in metal complexes. A higher absolute value of the binding energy indicates a more stable complex and a higher potential recognition capability of the ligand for the metal ions [53]. The binding energy for complexes involving N-heterocyclic carbene palladium, among others, is determined through the application of equation (1); This methodological approach not only quantifies the strength of the interaction but also provides insights into the structural stability and reactivity of these complexes.


XYW(R)=WXY(R)−WX(R)−WY(R) (1)

In equation (1), the term XYW(R) denotes the binding energy, while WXY (R) represents the overall energy of the interacting system. The energies of the two separate molecules, X and Y, are represented by WX (R) and WY(R) respectively. The binding energies associated with N-heterocyclic carbene palladium and various other complexes are detailed in Table 5. The specific binding energies for N-heterocyclic carbene ligands interacting with Pd, Pb, Hg, Cr, Cd, and As are quantified as −561.88, −373.05, −321.85, −480.61, −352.41, and −409.61 kJ/mol respectively. A clear observation from these data is that the binding affinity of N-heterocyclic carbene ligands with Pb, Hg, Cr, Cd, and As is significantly weaker compared to their interaction with Pd. Notably, the weakest binding is observed with Hg, while the binding energy with Cr exceeds that of all other heavy metals, excluding Pd. Compared with Cr, Pd has a higher electron affinity and a greater ionization energy, enabling Pd to form stronger coordination bonds with NHCs [54]. While compared with the remaining four heavy metal ions, the atomic radius of Cr is relatively tiny, and the bond length formed with N-heterocyclic carbenes is also shorter (consistent with the results shown in Table 1).

## 4. Conclusions

Palladium N-heterocyclic carbenes and their complexes with five hazardous heavy metals, namely Pb, Hg, Cr, Cd, and As, have been found to be stable entities. Remarkably, the complexes of N-heterocyclic carbenes with these heavy metals are actually more stable than the carbenes themselves. In terms of infrared and ultraviolet spectral absorption, these heavy metal-N-heterocyclic carbenes complexes exhibit distinct patterns from those of N-heterocyclic carbene palladium. This difference in absorption patterns allows for the qualitative differentiation of certain elements within these complexes from N-heterocyclic carbene palladium. However, the quantitative analysis of N-heterocyclic carbenes bound to Cr or As may present slightly more difficulties compared to those bound to Hg, Cd, or Pb. One interesting finding is that the complexes formed by Cr and N-heterocyclic carbenes ligands display the highest ultraviolet absorption intensity and chemical stability, This phenomenon may be attributed to the nitrogen heterocyclic carbene (NHC)-Cr complex adopting a tetrahedral geometry with Cr as the central atom. Regarding bond lengths, the C1-Cr bond length is comparable to that of the C1-Pd bond, whereas the N2-Cr bond length is shorter than the N2-Pd bond. The C1-Cr-N2 bond angle of 115.87168°closely approximates the ideal tetrahedral angle of 109.5°. By analyzing the Wiberg bond indices derived from Natural Bond Orbital (NBO) analysis, it is evident that the Wiberg Index values for the N-Cr and C-Cr bonds reach maximum values of 0.6274 and 0.8497, respectively. These findings collectively indicate that the coordination geometry of the Cr-NHC complex confers the highest degree of chemical stability. In terms of coordination binding capacity, among all the heavy metals tested, N-heterocyclic carbenes ligands exhibit the strongest affinity for Pd. These findings will undoubtedly provide valuable insights for future research on N-heterocyclic carbenes in the remediation of heavy metals, particularly in the targeted capture of Cr.
